# Assessing dynamical resilience indicators in older adults: a prospective feasibility cohort study

**DOI:** 10.1007/s41999-023-00904-9

**Published:** 2024-01-27

**Authors:** Daisy Kolk, Janet L. MacNeil Vroomen, René J. F. Melis, Milan L. Ridderikhof, Bianca M. Buurman

**Affiliations:** 1 Department of Elderly Care Medicine, Amsterdam UMC, Amsterdam Public Health Research Institute, Vrije Universiteit Amsterdam, De Boelelaan 1117, PO Box 7057, 1007 MB Amsterdam, The Netherlands; 2 Section of Geriatric Medicine, Amsterdam UMC, Internal Medicine, Amsterdam Public Health Research Institute, University of Amsterdam, Meibergdreef 9, Amsterdam, The Netherlands; 3 Department of Geriatric Medicine/Radboud Alzheimer Centre, Radboud University Medical Centre, Nijmegen, The Netherlands; 4 Amsterdam UMC, Emergency Medicine, Amsterdam Movement Sciences Research Institute, University of Amsterdam, Meibergdreef 9, Amsterdam, The Netherlands; 5 ACHIEVE-Center of Applied Research, Faculty of Health, Amsterdam University of Applied Sciences, Amsterdam, The Netherlands

**Keywords:** Resilience, Aged, Geriatrics, Older persons, Telemonitoring

## Abstract

**Purpose:**

Measuring dynamical resilience indicators based on time series data may improve the prediction of health deterioration in older adults after hospital discharge. We examined the feasibility of an intensive prospective cohort study examining dynamical resilience indicators based on time series data of symptoms and physical activity in acutely ill older adults who visited the Emergency Department (ED).

**Methods:**

This is a prospective cohort study with time series data from symptom questionnaires and activity trackers. Thirty older adults (aged 75.9 ± 5.5 years, 37% female) who were discharged from the ED of a tertiary hospital in the Netherlands were included in the study. We monitored self-reported symptoms using a daily online questionnaire, and physical activity using an activity tracker for 30 days. Descriptive data on participant eligibility and adherence to and acceptability of the assessments were collected.

**Results:**

Of 134 older patients visiting the ED, 109/134 (81%) were eligible for inclusion and 30/109 (28%) were included. Twenty-eight (93%) of the included participants completed follow-up. Regarding the adherence rate, 78% of participants filled in the questionnaire and 80% wore the activity tracker. Three (10%) participants completed fewer than three questionnaires. Most participants rated the measurements as acceptable and seven (23%) participants experienced an adverse outcome in the 30 days after discharge.

**Conclusion:**

Such an intensive prospective cohort study examining dynamical resilience indicators in older adults was feasible. The quality of the collected data was sufficient, some adjustments to the measurement protocol are recommended. This study is an important first step to study resilience in older adults.

## Key summary points

**Aim:**

To examine the feasibility of a prospective cohort study examining dynamical resilience indicators in acutely ill older adults who visited the Emergency Department (ED).

**Findings:**

The intensive examination of dynamical resilience indiciators based on symptoms and physical activity measurements is feasible in older ED patients.

**Message:**

The method of intensive dynamical resilience indicators assessment can be used to study resilience in older ED patients.

## Introduction

Older adults are living longer and have more chronic disabilities, which increases their need for acute care. In the Netherlands, approximately 800,000 older adults visit the emergency department (ED) each year and this number is expected to increase by 40% in the coming years [1, 2]. Acute medical events that require admission to an ED are stressful for older adults, and this increases their risk of adverse health outcomes after discharge [3–5]. Up to 20% of older ED patients have to revisit the ED unexpectedly within 1 month after discharge from the ED [4]. Risk factor assessment and screening tools can be used to estimate the risk of these adverse outcomes before discharge [3–6]. However, these prognostic assessments do not sufficiently predict who will recover after ED discharge and who will need to be readmitted [7–9]. Assessing physical resilience [10], particularly early dynamical indicators of resilience [11, 12], after discharge provides opportunities to predict recovery and timely detect critical declines in health that may lead to readmission in community-dwelling older adults.

Physical resilience is defined as an individual’s capacity to resist or recover from functional decline after a health stressor such as acute illness and subsequent hospitalization [10]. Several researchers have argued that recovery from a stressor is dynamic [13–15], so should be assessed with continuous measurements. According to the complex systems theory, dynamic parameters tend to slow down in a complex system and become closely correlated when resilience is lost and a critical transition approaches [16]. Using this theory, researchers in the field of depression [17] have collected time series data on symptoms in individual patients using telemonitoring. Symptoms were monitored on a daily basis and found that indicators of slowing down, like a higher variance in symptoms predicted a critical transition to depression. This approach including physical activity measurements improved the prediction of recovery in hospitalized older patients [18–22]. However, no previous studies investigated whether resilience indicators can also be found in time series data on symptoms and physical activity in community-dwelling older adults after acute health stressors.

We hypothesize that this approach of monitoring dynamical resilience indicators over time may also improve prediction of recovery in older adults discharged from the ED. Time series data have not been collected from older patients after ED discharge yet, so our first aim was to examine the feasibility of a prospective cohort study collecting time series data and intensive longitudinal data (ILD) on symptom burden and physical activity from older individuals for 30 days after ED discharge. We evaluated institution-based feasibility, patient-based feasibility, and acceptability of the assessments. The second aim of this study was to investigate the individual trajectories of symptom burden and physical activity over time.

## Methods

### Setting and participants

We conducted a prospective feasibility cohort study at the ED of a tertiary hospital in the Netherlands from October 2019 to January 2020. All patients age 70 years and over who were discharged from the ED to an independent living arrangement were screened for inclusion. Patients were excluded if they (1) were diagnosed with dementia, or (2) were not able to complete the questionnaires in Dutch. The treating physician checked the eligibility criteria. Trained researchers approached eligible patients before discharge. A sample of 30 participants was considered sufficient to examine feasibility.

All participants were asked for written informed consent. This study was approved by the institutional review board (Protocol ID: 2019_186) and was performed according to the Dutch Medical Research Involving Human Subjects Act and principles of the Declaration of Helsinki (1964).

### Data collection

Trained researchers collected baseline data by reviewing charts and assessing patients. Baseline assessments included demographics; main practicing medical specialty; number of morbidities; the Charlson Comorbidity Index (CCI) [23]; polypharmacy; and pre-morbid number of disabilities in activities of daily living (ADLs) [24]. Dynamical resilience indicators were based on a time series of self-rated symptom burden and physical activity [18, 19]. Symptom burden was assessed once a day for 30 days after discharge using an online questionnaire. The questionnaire included a modified Edmonton Symptom Assessment Scale [25, 26] of 13 physical and emotional symptoms that were scored on a 11-point numeric rating scale from 0 (no burden) to 10 (high burden). These symptoms included pain, difficulty swallowing, difficulty with bowel movements, nausea, dyspnea, dizziness, sleeping problems, poor appetite, low energy, fatigue, anxiety, gloom, and low well-being. If participants were not able to complete the questionnaire online, they were contacted by telephone. A total symptom burden score was calculated as the sum of all symptoms (range 0–130). Physical activity (i.e., the number of steps taken) was continuously measured for 30 days after discharge using the Fitbit Flex activity tracker [27, 28]. Participants were asked to wear the tracker continuously and to charge it every 7 days. On day 30, the researcher visited the participants to collect the tracker. Data were also collected on the participant’s study experiences, healthcare use, and adverse outcomes. Adverse outcomes included mortality, revisits, and functional decline. Functional decline was defined as having more disabilities in ADLs [24] 30 days after discharge than pre-morbid 2 weeks prior to the ED admission.

### Feasibility and acceptability measures

The primary outcomes were institution-based feasibility (measured as the eligibility rate) and patient-based feasibility (measured as the inclusion rate and adherence rate). Data were collected on the number of patients screened, the number of eligible participants, reasons for non-participation, and adherence to the study protocol. The eligibility rate was calculated as the number of eligible patients divided by the number of patients who were discharged home. The inclusion rate was calculated as the number of inclusions divided by the number of eligible patients. The adherence scores for completing the questionnaire and wearing the activity tracker were calculated as the total number of days the questionnaires were filled in/the activity tracker was worn, divided by the number of follow-up days per participant and then averaged at group level. For the questionnaire, the individual adherence score per patient and the adherence score per day were calculated.

The secondary outcomes were acceptability of the study processes and representativeness of the study sample. Acceptability was assessed by a questionnaire at the end of study. Representativeness was assessed by comparing age, sex, and medical specialty of the study cohort with those of the complete population of older patients discharged from the ED.

### Statistical analysis

We used descriptive statistics to describe eligibility rate, inclusion rate, adherence rate, acceptability, symptom burden, and physical activity. Normally distributed continuous variables were expressed as mean and standard deviation (SD) and non-normally distributed variables were expressed as median and interquartile range (IQR). Categorical variables were presented as numbers and percentages. Cohort representativeness was examined using chi-square tests for categorical variables and Wilcoxon rank-sum tests for non-normally distributed continuous variables.

To study the trajectories of symptom burden and physical activity, we modeled the individual changes over time. We used multilevel regression analysis with individual growth modeling [29] to describe the individual changes in symptom scores and physical activity over time. While performing longitudinal multilevel regression models [30] we did not impute the missing values in the dependent variable using a multiple imputation technique [31]. In our model, imputations of the missing values are based on the likelihood. For each model, we started with an unconditional linear growth model. Based on the time series, visualized using graphs, we constructed more complex models of the data as a non-linear function by adding restricted cubic splines [32]. Using the deviance statistic, we explored which model best explained the changes over time.

We also tested whether changes in single symptoms correlate with changes in other symptoms in an individual. We fitted a linear growth model for each symptom and extracted the variances over time per individual. Correlations between the variances of single symptoms were assessed with the Spearman rank correlation coefficient. Descriptive analyses were performed in SPSS (Version 28.0; SPSS, Inc., Chicago, IL) and multilevel regression analyses were performed in RStudio/1.3.1093 (RStudio Team (2020), RStudio: Integrated Development for R. RStudio, Inc., Boston, MA, URL http://www.rstudio.com/). The multilevel regression models were fitted in “lme4”.

## Results

### Eligibility rate and inclusion rate

**Fig. 1:**
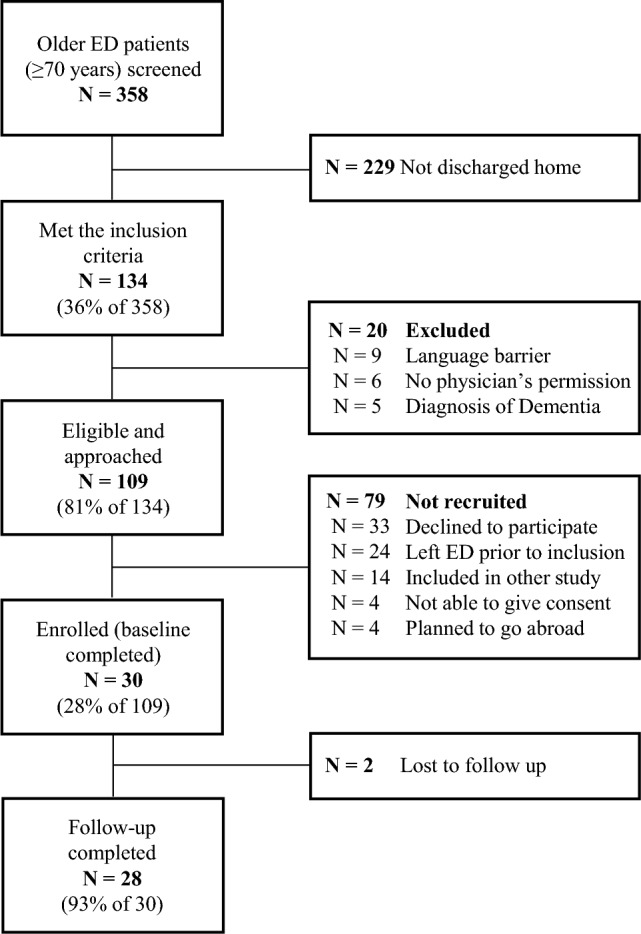
Flowchart of inclusion and follow-up of participants. *Emergency department

Of the 2132 patients that visited the ED during the study period, 358 were 70 years old or older and further screened for eligibility. Of these patients, 134 (36%) were discharged home and 109/134 (81%) of these were eligible and approached for participation. Eventually, 30/109 participants (28%) were included in the study (Fig. 1). The main reasons for not participating were lack of energy after the long ED stay and considering the study protocol too intensive. Participant characteristics are described in Table 1. Participants had a mean (SD) age of 75.9 (5.5) years, 11 (37%) were female, and 14 (47%) had multi-morbidities.

**Table 1:** Descriptive characteristics of the participants

Patient characteristics	*N* = 30
Age, mean (SD), years	75.9 (5.5)
Range	70–92
Female, *n* (%)	11 (37)
Education, *n* (%)	
Primary/elementary technical/domestic school	7 (23)
Secondary vocational education	7 (23)
Higher level high school/third-level education	16 (54)
Marital status, *n* (%)	
Single/divorced	7 (23)
Married	20 (67)
Widowed	3 (10)
Living situation, *n* (%)	
Alone	10 (33)
Living with partner	20 (67)
Main practicing specialism at ED, *n* (%)	
Neurology	9 (30)
Emergency medicine	7 (23)
Cardiology	4 (14)
Surgery	3 (11)
Gastroenterology	2 (7)
Internal medicine	1 (3)
Geriatrics	1 (3)
Pulmonology	1 (3)
Neurosurgery	1 (3)
Ophthalmology	1 (3)
Multi-morbidity, *n* (%)^a^	14 (47)
Charlson comorbidity Index, median (IQR)^b^	1 (0–2)
Range	0–7
Polypharmacy, *n* (%)^c^	15 (50)
Katz-ADL score premorbid, median (IQR)^d^	0 (0–0)
Range	0–3

*ED* emergency department, *SD* standard deviation, *IQR* interquartile range, *ADL* activities of daily living

^a^Two or more chronic illnesses in the medical history

^b^Range of 0–31, with a higher score indicating more or severe comorbidity

^c^Use of 5 or more different medications

^d^Ranging from 0 (independent at all ADLs) to 6 (dependent on all ADLs)

### Adherence rate

Of all participants, 28 (93%) completed follow-up. One patient was lost to follow-up after 13 days, and one patient was readmitted after 9 days and withdrew from the study. Accumulated total study follow-up was used as the denominator to calculate the adherence score, resulting in 862 follow-up days.

The symptom questionnaire was filled in completely on 673/862 follow-up days, resulting in an overall adherence rate of 78%. The median (IQR) individual adherence to completing the symptom questionnaire was 97% (70–100). Twenty-one patients (70%) completed the questionnaire on more than 80% of follow-up days. Three patients (10%) completed the questionnaire on less than 10% of follow-up days as they found it too troublesome with their medical condition. Twenty-four percent of questionnaires were filled in by telephone (with help from a researcher) instead of online. Reasons for needing assistance were (1) not having an email address, (2) vision problems, and (3) computer problems.

All 30 participants agreed to wear the activity tracker and 24 (80%) eventually wore the Fitbit. Three participants (10%) could not wear it for medical reasons, three (10%) for logistic reasons, and one lost the Fitbit. Data were available from 23 participants over 669 follow-up days. The Fitbit was worn on 534/669 days (adherence rate 80%). Of the 24 patients who wore the Fitbit, six (25%) said they were unable to load the battery successfully, which lowered the adherence rate.

### Acceptability and representativeness

Of the 28 participants who completed follow-up, five (18%) said filling in the symptom questionnaire every day was troublesome. Of the 22 patients who filled in the questionnaire online, 16 (73%) said the questionnaire was user-friendly. Wearing the activity tracker was rated as troublesome by 2/24 (8%) participants.

Age of participants was comparable to that of the general older ED population who were discharged home, but more individuals in the study cohort were male than those in the general ED population were. More patients were treated by a neurologist or cardiologist while fewer patients were treated by an internist.

### Adverse outcomes, symptoms, and physical activity

**Fig. 2:**
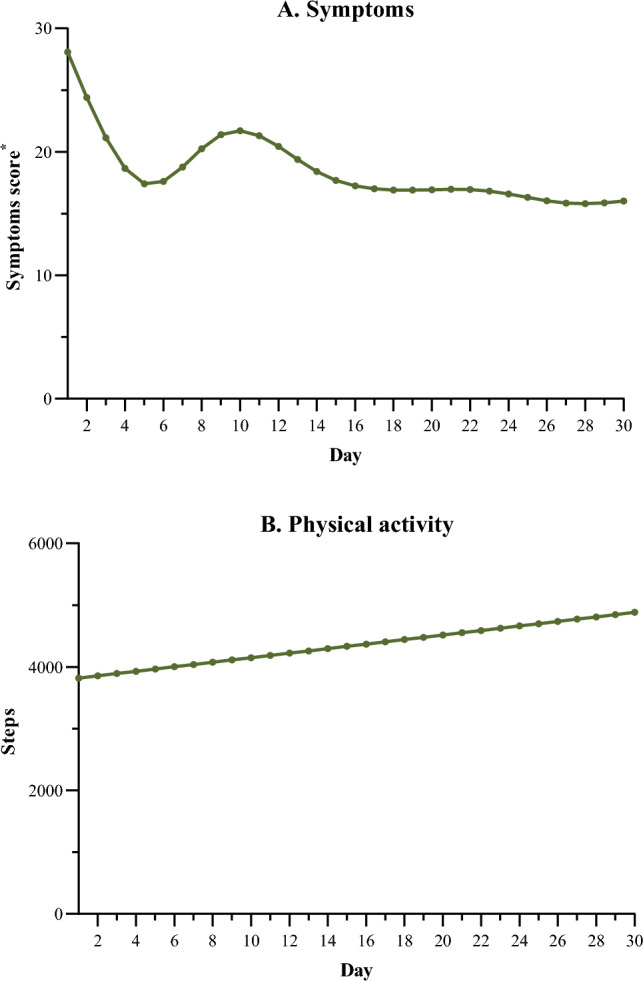
Predicted mean growth curves of individual changes in symptom burden (**A**) and physical activity (**B**) modeled over time for 30 days after discharge using multilevel regression analysis. *The total symptom score is calculated as the total sum of all 13 symptoms assessed on a numeric rating scale (range 0–10). The total symptom score ranges from 0 to 130, higher scores indicating a higher overall symptom burden

Four participants (13%) had an unplanned revisit and three participants (10%) experienced functional decline within 30 days after discharge. The median (IQR) number of symptoms was 5 (1–7) during the 30 days after discharge. The number of symptoms remained stable from 1 day after discharge (median 5; IQR 3–8) to 30 days after discharge (median 5; IQR 0–7). The total median (IQR) symptom score (range 0–130) was 15 (2–28) 30 days after discharge. The median (IQR) symptom score was highest 1 day after discharge (25 [9–47]) and reduced to 15 (0–26) 30 days after discharge. The individual symptom scores fluctuated over time and were best described with a natural cubic spline. The mean growth curve of the symptom scores predicted by this model is shown in Fig. 2A. We also evaluated within-person variances in symptom score for each symptom and found high correlations between poor appetite and sleeping problems, low energy and fatigue, and fatigue and low well-being (*r* =  > 0.80, *P* < 0.001).

The overall median (IQR) number of steps was 4205 (1706–7145). On the first day after discharge, the median (IQR) number of steps was 3091 (963–6128) and this increased to 5608 (2325–8400) steps at 30 days after discharge. Participants with at least one disability in ADLs took a median (IQR) number of 1338 (745–1925) steps after discharge. When modeling step numbers over time, we found that a model with a linear growth curve best described the individual changes in step numbers. Figure 2B shows the mean growth curve of steps taken predicted by this model.

## Discussion

We explored the eligibility, inclusion, adherence, and acceptability of a prospective cohort study collecting time series data on symptom burden and physical activity in community-dwelling older adults for 30 days after discharge from the ED. This study seemed sufficiently feasible and acceptable for a larger scale study and revealed important implications for the design of a larger scale study. The data were of sufficient quality to model the time series and extract dynamical resilience indicators.

We have shown that the dynamical resilience indicators approach is a feasible way to monitor recovery in community-dwelling older adults after major health stressors [17, 19]. This approach may improve the prediction of recovery in older patients after discharge from the ED. Most older patients are discharged home from the ED even though they have a high risk of insufficient recovery [33]. Monitoring symptoms and physical activity every day was acceptable to most participants in our study, but was too intensive for the more frail participants. Therefore, to provide generalizable results, a larger scale study needs to include a representative sample of older adults that includes individuals who are more frail.

Our study also revealed implications about patient recruitment in a larger scale study. The inclusion rate and proportion of patients that declined to participate in our study were similar to those of a previous similar study [7]. In our study, many patients were eligible for participation but were discharged before informed consent could be obtained. In addition, many eligible patients declined to participate because they did not feel well enough to stay in the ED. The ED is a stressful place to be and most patients are happy to be discharged from this uncertain environment [34]. Our findings suggest that more patients could have been recruited if the inclusion period were extended, e.g., digitally within the first week after discharge [7].

We also examined adherence rates in our study. To our knowledge, no previous studies have collected time series data on a daily basis from community-dwelling older adults after an ED visit, so we wanted to determine how well participants would adhere to this approach. Our study had lower adherence scores than another study that assessed time series data in older adults [19], but this may be because participants were assisted with their measurements every day in the other study. Bongers et al. [35] also collected time series data from community-dwelling older adults and reported similar adherence scores to ours.

We found an inclusion rate of 28%, which suggests that selection bias has occurred. Some of the patients and caregivers we approached said that the study would be too intensive and declined to participate, which suggests that especially more frail older persons were not included in the study. This also highlights the need for user-friendly, passive measurement tools in a larger scale study. This is supported by our finding that participants adhered more to wearing the activity tracker than to filling in the questionnaires and that participants reported the questionnaires to be more burdensome than the activity tracker. Despite this, adherence rates for completing the questionnaires and wearing the activity tracker were high enough to model these data over time and to extract within-person variances as an indicator of resilience [11, 12, 16, 19]. Only 10% of the patients did not feel well enough to fill in questionnaires for at least three days, which should be taken into consideration when calculating how many participants to recruit in a larger-scale cohort study. Another important finding was that 25% of questionnaires were filled in by telephone with help from a researcher. This makes the study more time-consuming for researchers and advocates user-friendly sensor technology instead of online questionnaires [36].

We also showed how the time series on symptoms and physical activity can be modelled using individual growth models. Using these models we have predicted the mean growth curves. We found that the symptom data could best be modelled using a nonlinear model, showing a peak in symptom score at day 10. We also found that physical activity could best be modelled using a linear model and that the physical activity levels increased from discharge over time, which was also found in a previous study with acutely hospitalized older patients [37]. We hypothesize that the initial decline in physical activity already took place before the patient was admitted to the ED. However, we do not have baseline measurements prior to admission and we have included a too small sample size to investigate these trajectories precisely. In a larger cohort study, the time series can be modelled using individual growth models with less uncertainty.

### Strengths and limitations

In this study, we collected time series data on symptom burden and physical activity in older patients for 30 days after discharge from the ED. Our findings reveal the feasibility of collecting time series data on dynamical resilience indicators in a heterogeneous group of community-dwelling older adults after discharge from the ED. These findings will be useful for designing other studies. In our sample of older ED patients, women and patients treated by an internist were underrepresented, which would reduce the generalizability of the results in a large-scale cohort study. In addition, we were not able to assess whether selection bias occurred with regard to frailty status or other patient characteristics. Due to privacy reasons, we were not allowed to collect data of patients that were eligible but did not consent to participate. Moreover, this study was only conducted in one university medical center, which may reduce the generalizability of the results to patients visiting a general hospital. A multicenter cohort study in general hospitals should be feasible as the study population probably has less-complex medical problems.

### Conclusions and implications

A prospective cohort study that collects time series data on dynamical resilience indicators from community-dwelling older adults for 30 days after ED discharge is feasible and acceptable. However, some adjustments to the study protocol are recommended. To improve inclusion rates, the recruitment period should be extended for one week after ED discharge. To increase adherence to the protocol, user-friendly sensor technology should be used. These findings are informative for future studies on dynamical resilience indicators in community-dwelling older adults.
